# Supplementary material for: Evaluating the Implementation of a Multicomponent Intervention Consisting of Education and Feedback on Reducing Benzodiazepine Prescriptions by General Practitioners: BENZORED Hybrid Type I Cluster Randomized Controlled Trial
Source: Int J Environ Res Public Health. 2021 Jul 28;18(15):7964. doi: 10.3390/ijerph18157964 (PMC8345522; doi:10.3390/ijerph18157964)
Supplement: Supplementary file 1 [file ijerph-18-07964-s001.zip › ijerph-1239819-supplementary.pdf]

International Journal of *Environmental Research and Public Health*

MDPI

Table S1. Focus group discussion guide.

**0. Implementation**	**What is your overall opinion of the implementation of the BENZORED intervention at your center?**
**I. Intervention characteristics**	**What do you think of the intervention and which of its characteristics do you think influenced its implementation?**
A. Intervention source	How did knowing that the intervention had been designed by general practitioners like you influence your participation?
B. Evidence strength and quality	How do you feel about the fact that the intervention was based on scientific evidence?
D. Adaptability	What do you think about the adaptability of the intervention? Which aspects did you adapt to your center?
G. Design quality and packaging	What do you think about the training received? And the materials provided?
**II. Healthcare system (outer setting)**	**How do you feel about the fact that the intervention is being implemented in other settings (other autonomous communities, other parts of the words, inclusion in clinical guidelines)**
A. Peer pressure	
D. External policies and incentives	How did the existence of a primary care management–level quality indicator for measuring chronic benzodiazepine (BZD) use influence you?
**III. Healthcare center (inner setting)**	**Which aspects of your healthcare center do you think facilitated the implementation of the BENZORED intervention? And which acted as a barrier?**
D.1. Tension for change	Do you think that high BZD use among the patients under your care is a problem that needed to be addressed? Why?
D.3. Relative priority	How would you prioritize the BENZORED intervention in relation to other interventions or initiatives at your center?
E. Readiness for implementation	How willing do you think your center is to incorporate new ideas or improvements that involve changing existing ways of working?
**IV. Characteristics of individuals**	**Which personal characteristics do you think facilitated the implementation of the BENZORED intervention?**
A. Knowledge and beliefs	How effective do you think the intervention is in terms of reducing BZD use? How does it compare to what used to be done?
B. Self-efficacy	Now that you have participated in the project, how do you feel about your ability to prescribe BZDs properly and to implement withdrawal strategies?
**V. Process**	**What do you think about the process (planning, execution, and evaluation) used to integrate the BENZORED intervention into your practice?**
A. Planning	Did the project go as planned at your center? Were any changes introduced with respect to training?
D.6. Learning climate	What do you think about the periodic feedback you have received regarding your BZD prescribing habits? How did seeing differences between what you do and what others in your group do influence you? Do you feel you have had a key role in reducing BZD use?
B.1. Opinion leaders	How do you think the project management team influenced you and the implementation of the intervention?
D. Reflecting and evaluating	What do you think could be done to improve the implementation of the intervention? (feasible solutions)

Table S2. GPs proposal for implementation.

**Planning**	1- Implement clinical practice guidelines on the proper use of benzodiazepines (BZDs) in primary care settings and hospitals.<br>2- Engage primary care pharmacists to help identify chronic BZD users and alert general practitioners (GPs).<br>3- Implement community interventions on appropriate BZD use in nursing homes, civic centers, caregiver training centers, psychoeducational groups…
**Educating**	1- Use the media to increase awareness of the adverse effects of chronic BZD use.<br>2- Distribute posters and leaflets in primary healthcare centers.<br>3- Place brief reminders about the intervention on GPs' desks.<br>4- Promote appropriate BZD use through patient safety (deprescription) and chronic disease self-management programs.<br>5- Provide GPs with regular feedback about their BZD prescribing habits in comparison to those of other GPs and primary healthcare centers.
**Financing**	1- Develop a computerized support system including reminders, pre- and post- BZD prescription advice, and chronic BZD use alerts in the electronic prescription system.<br>2- Compile lists of chronic BZD users in the care of each GP and schedule longer visits to assess BZD withdrawal in these patients.<br>3- Include intervention materials in patients' medical records or on GPs' computers.
**Restructuring**	1- Involve primary care nurses. Train them on caring for patients with sleeping difficulties and providing sleep advice prior to the prescription of BZDs. Heighten their awareness of the need to be attentive to frail patients with frequent falls possibly related to BZD use.
**Managing quality**	1- Assign a go-to member of the research team at each primary healthcare center. GPs appreciate the proximity of the research group and knowing who they can go to to resolve doubts.<br>2- Designate a BENZORED champion as a liaison at each primary healthcare center to organize periodic meetings to review the success of the intervention, recall important points, resolve doubts, and share experiences.
**Attending to policy context**	None

GP proposals are summarized in the table below and classified by the six key processes defined by Powell et al. (2012).
